# RNA-binding proteins that are highly expressed and enriched in healthy cartilage but suppressed in osteoarthritis

**DOI:** 10.3389/fcell.2023.1208315

**Published:** 2023-06-30

**Authors:** Hannah Swahn, Merissa Olmer, Martin K. Lotz

**Affiliations:** Department of Molecular Medicine, Scripps Research, La Jolla, CA, United States

**Keywords:** RNA-binding proteins (RBPs), osteoarthritis (OA), cartilage, TCDD inducible poly(ADP-ribose) polymerase (TIPARP), RNA-sequencing (RNA-seq), genotype-tissue expression (GTEx), single cell RNA-sequencing (scRNA-seq)

## Abstract

**Objectives:** RNA-binding proteins (RBPs) have diverse and essential biological functions, but their role in cartilage health and disease is largely unknown. The objectives of this study were (i) map the global landscape of RBPs expressed and enriched in healthy cartilage and dysregulated in osteoarthritis (OA); (ii) prioritize RBPs for their potential role in cartilage and in OA pathogenesis and as therapeutic targets.

**Methods:** Our published bulk RNA-sequencing (RNA-seq) data of healthy and OA human cartilage, and a census of 1,542 RBPs were utilized to identify RBPs that are expressed in healthy cartilage and differentially expressed (DE) in OA. Next, our comparison of healthy cartilage RNA-seq data to 37 transcriptomes in the Genotype-Tissue Expression (GTEx) database was used to determine RBPs that are enriched in cartilage. Finally, expression of RBPs was analyzed in our single cell RNA-sequencing (scRNA-seq) data from healthy and OA human cartilage.

**Results:** Expression of RBPs was higher than nonRBPs in healthy cartilage. In OA cartilage, 188 RBPs were differentially expressed, with a greater proportion downregulated. Ribosome biogenesis was enriched in the upregulated RBPs, while splicing and transport were enriched in the downregulated. To further prioritize RBPs, we selected the top 10% expressed RBPs in healthy cartilage and those that were cartilage-enriched according to GTEx. Intersecting these criteria, we identified Tetrachlorodibenzodioxin (TCDD) Inducible Poly (ADP-Ribose) Polymerase (TIPARP) as a candidate RBP. TIPARP was downregulated in OA. scRNA-seq data revealed TIPARP was most significantly downregulated in the “pathogenic cluster”.

**Conclusion:** Our global analyses reveal expression patterns of RBPs in healthy and OA cartilage. We also identified TIPARP and other RBPs as novel mediators in OA pathogenesis and as potential therapeutic targets.

## Introduction

Osteoarthritis (OA) is an age-related condition that is becoming more prevalent with a quickly aging world population. Knee OA accounts for a substantial fraction of the overall prevalence, impacting patient mobility and quality of life. A major mechanism of OA pathogenesis is the dysregulation of gene expressions in the affected tissues. Recent RNA-sequencing studies in human and rodent OA knees revealed global dysregulation in genes involved in extracellular matrix (ECM) components (Periostin (*POSTN*), Collagen Type I Alpha 1 Chain (*COL1A1*), Collagen Type X Alpha 1 Chain (*COL10A1*)), ECM-degrading proteinases (ADAM Metalloproteinase with Thrombospondin Type 1 Motif 5 (*ADAMTS5*), Matrix Metalloproteinase 13 (*MMP13*), etc.), circadian rhythm pathways (Basic Helix-Loop-Helix ARNT Like 1 (*BMAL1*)) and mechanotransduction (Piezo Type Mechanosensitive Ion Channel Components 1 and 2 (*PIEZO1* and *PIEZO2*), Transient Receptor Potential Cation Channel Subfamily V Member 4 (*TRPV4*), etc.) (Dudek et al., 2016; Fisch et al., 2018; Hu et al., 2023).

Therapeutic targets have often been selected for their role in cartilage damage and inflammation. RNA-binding proteins (RBPs) are a class of proteins that are involved in the regulation of gene expression, as well as post-transcriptional processes via binding of RNA molecules (Gerstberger et al., 2014). Therefore, RBPs play critical roles in cell homeostasis and tissue development, and are often altered in cancers, inflammatory and age-related diseases such as OA. RBPs function mostly through regulation of RNA metabolism, which includes a variety of processes such as RNA synthesis (Sun et al., 2006; Hata et al., 2008; Gu et al., 2014; Shanmugaapriya et al., 2016; Deng et al., 2019; Zhu et al., 2021), alternative splicing (Ni et al., 2021; Shen et al., 2021), RNA stability (Nieminen et al., 2008; McDermott et al., 2016; Son et al., 2019; Bai et al., 2020; Chen et al., 2022; He et al., 2022; Xiao et al., 2022), RNA transport/localization (Ansari and Haqqi, 2016; Huang et al., 2017; Chang et al., 2021) and translation (Takagi et al., 2005; Niu et al., 2017; Li et al., 2019; Deng et al., 2020). However, although it is suggested that RBPs can contribute to OA pathogenesis, a global analysis of RBPs in healthy and OA cartilage is yet to be reported.

The objectives of this study were to (i) map the global landscape of RBPs expressed and enriched in heathy cartilage and dysregulated in OA; (ii) prioritize RBPs for their potential role in cartilage. Our data identify Tetrachlorodibenzodioxin (TCDD) Inducible Poly (ADP-Ribose) Polymerase (TIPARP) as a candidate RBP for future studies, and a potential novel therapeutic target for OA cartilage.

## Methods

### Previously published datasets utilized: RNA-sequencing (RNA-seq), Genotype-Tissue Expression (GTEx) and single cell RNA-sequencing (scRNA-seq)

Data from our previously published bulk RNA-sequencing (RNA-seq) analysis of normal (*n* = 18) and OA (*n* = 20) human cartilage samples were utilized in this study (Fisch et al., 2018). RBPs were considered differentially expressed (DE) in OA cartilage compared to healthy, if the adjusted *p*-value was less than 0.05. We also set an average RNA-seq counts cutoff of >100 in healthy cartilage to eliminate RBPs that were not expressed at biologically significant levels.

As described in (Gamini et al., 2017; Lee et al., 2020), the RNA-seq data from healthy human knee cartilage (*n* = 15) were compared with similar data from 37 tissues with a minimum of 3 unique samples and a maximum of 25 samples in the Genotype-Tissue Expression (GTEx) database, resulting in a total of 699 samples and 38 tissues included in the analysis. Cartilage donor ages ranged from 18 to 57 years (mean age 32.67 ± 10.82). For all other tissues, the donor ages ranged from 20–49 (in most tissues the largest % of donors ranged from 40–49) (Supplementary Figure S1A). Cartilage donors included 11 males and 4 females, similar to a larger number of males in GTEx (Supplementary Figure S1B). From this analysis, 313 genes were shown to be cartilage-enriched (Supplementary Table S1), and we utilized these genes in our present study.

Finally, data from our previously published single cell RNA-sequencing (scRNA-seq) analysis of healthy (*n* = 6) and OA (*n* = 6) human cartilage samples were utilized in this study (Swahn et al., 2023). Cluster-specific differential *TIPARP* expression was determined between healthy and OA. The largest differential expression (based on log_2_FC) was observed in the “pathogenic cluster”, which was so termed due to enrichment of genes involved in OA pathogenesis.

### RNA-binding proteins (RBPs), transcription factors (TFs), long non-coding RNAs (lncRNAs) and extracellular matrix (ECM) protein categories

**FIGURE 1:**
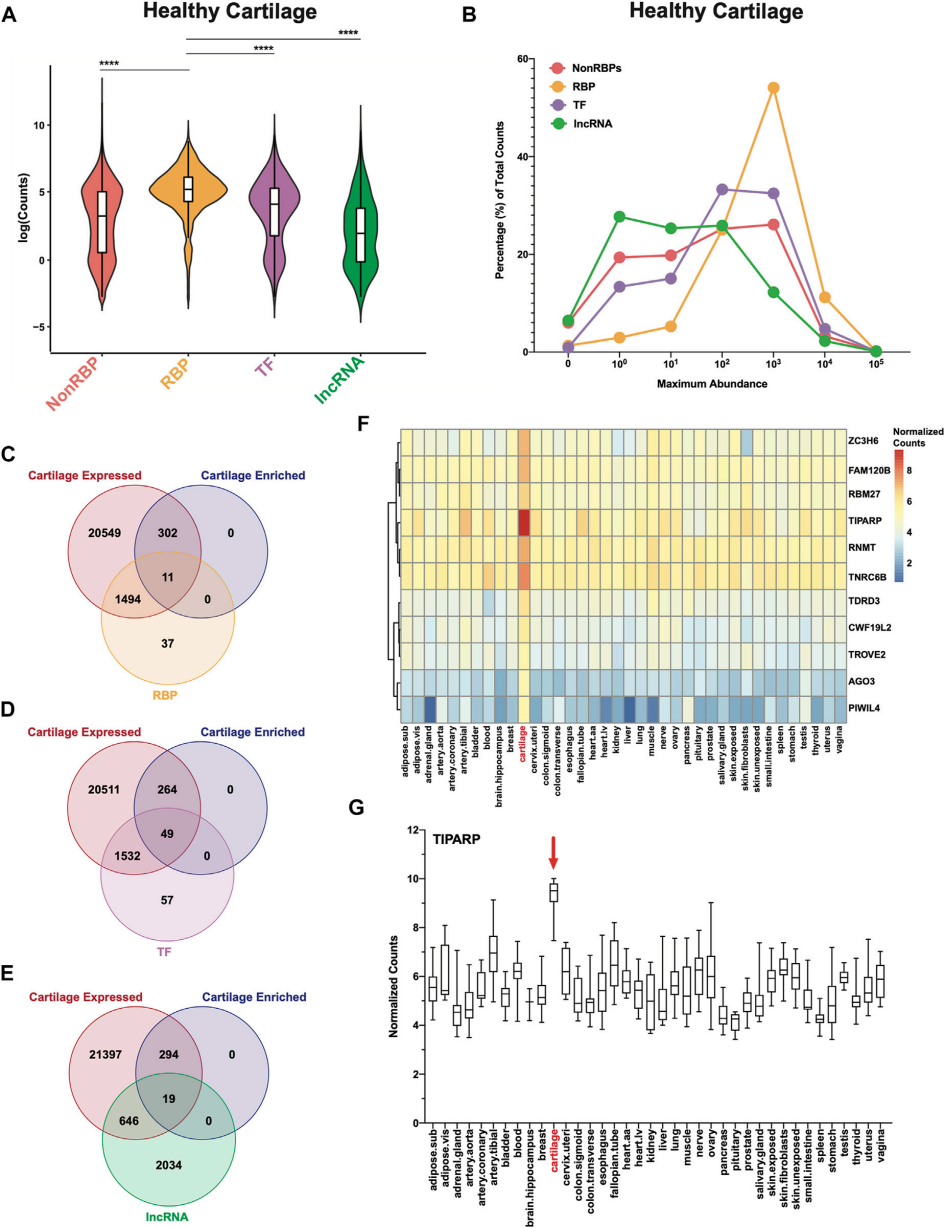
RBPs are highly expressed and enriched in healthy human cartilage. **(A)** Log normalized RNA-seq counts for nonRBPs, RBPs, TFs and lncRNAs in healthy cartilage (*n* = 18). Box and whiskers plots showing the first and third quartiles. Center line at the median. Wilcoxon rank sum tests with continuity correction were used to determine statistical significance between expression of RBPs and all other categories. ****p < 2.2e-16. **(B)** Maximum abundance levels of nonRBPs, RBPs, TFs and lncRNAs are shown as percentage of total RNA-seq counts for each category. **(C–E)** Intersection of cartilage expressed genes (22,356; RNA-seq counts >0), GTEx cartilage-enriched genes (313) and RBPs **(C)**, TFs **(D)** and lncRNAs **(E)**. **(F)** Heatmap showing average normalized counts of cartilage-enriched RBPs compared to all other GTEx tissues. **(G)** TIPARP normalized counts for all samples across all GTEx tissues. Box and whiskers plot showing the first and third quartiles. Center line at the median. For all panels, cartilage bulk RNA-seq data from (Fisch et al., 2018).

A census of 1,542 known RBPs (Gerstberger et al., 2014), 1,638 known transcription factors (TFs) (Lambert et al., 2018) and 2,699 long non-coding RNAs (lncRNAs) from the GENCODE project (Derrien et al., 2012; Harrow et al., 2012) were utilized in this study (Supplementary Table S2). The ‘nonRBP’ category shown in Figure 1 includes all genes expressed in healthy cartilage except RBPs. ECM categories were derived from the Naba lab (University of Chicago) and are available on MatrisomeDB (https://matrisomedb.org/) (Shao et al., 2020; Shao et al., 2023) (Supplementary Table S2).

### Protein-protein interaction (PPI) network analyses

Protein-protein associations of the DE RBPs in OA cartilage were investigated using the STRING database (v11.5) (Szklarczyk et al., 2011). We input our list of DE RBPs and selected interactions pertaining only to *Homo sapiens*. We included only interactions with a minimum confidence score of 0.4. To simplify the complex PPI network, we then clustered the interactions using the STRING *k*-Means clustering algorithm (MacQueen, 1967). We chose 10 clusters, as this number was determined using the rule of thumb (*k* = **√** *n*/2) (Mardia et al., 1979), where *n* is equal to the number of nodes (protein interactors, *n* = 188) in the network and *k* is the number of clusters of interest. The clusters were then separated and exported into Illustrator (Adobe v3.0) for visualization.

### Gene ontology and biological pathways analyses

Gene ontology analyses were performed on the DE RBPs using gProfiler (Raudvere et al., 2019). Biological pathway analyses were performed on the DE RBPs using Kyoto Encyclopedia of Genes and Genomes (KEGG) (Kanehisa and Goto, 2000; Kanehisa, 2019; Kanehisa et al., 2023). The up- and downregulated RBPs were separately analyzed with gProfiler and KEGG. Bar charts generated using GraphPad Prism (v8.4.2) were used to visualize the gProfiler results. Radar plots generated using the Functions for Medical Statistics Book with some Demographic Data (fmsb, v0.7.5) package in R software (v4.1.1) were used to visualize the KEGG results.

### ChIP-X Enrichment Analysis 3 (ChEA3): transcription factor enrichment analyses

To determine putative TFs that regulate the DE RBPs in OA cartilage, we used ChIP-X Enrichment Analysis 3 (ChEA3) (Keenan et al., 2019). The publicly available web-server can be accessed from: https://amp.pharm.mssm.edu/ChEA3. ChEA3 makes predictions of enriched TFs for a user-provided list of genes. These predictions are made from a variety of assays and other evidence sources contained within the ChEA3 database including: i) TF-gene co-expression patterns from RNA-seq experiments, ii) TF-target associations from chromatin immunoprecipitation (ChIP)-seq studies and iii) TF-gene co-occurrences determined by user-inputted gene lists. The “Mean Rank Scores”, which is the mean rank of the TF across all libraries in the ChEA3 database, were used to rank the enriched TFs in the lists of up- and downregulated RBPs. Cytoscape (v3.9.1) was used to visualize the TF-RBP networks.

### Immunohistochemistry (IHC)

Four μM sections were cut from healthy and OA human knee cartilage. Sections were deparaffinized, rehydrated and washed. Blocking was performed using Bloxall Solution (Vector Laboratories), then 2.5% Normal Horse Serum for 1 h at room temperature. Sections were incubated with TIPARP, shown in the Table 1 overnight at 4°C. Rabbit IgG (Vector Laboratories) was used as negative control.

**TABLE 1:** TIPARP IHC conditions.

Antibody	Supplier	Cat#	Host	Retrieval method	Primary dilution
TIPARP	Fisher Scientific	PA598589	Rabbit	None	1:300

For IHC of TIPARP, the sections were washed and incubated with biotinylated anti-rabbit IgG for 1 h at room temperature and then incubated with VectaElite ABC kit (Vector Laboratories). All sections were then stained with DAB staining. Counterstains were done with methyl green and/or hematoxylin. Quantification of positive cells in IHC human cartilage tissues, areas within 700 μm from the articular surface were included in the analysis. Two areas were counted at ×10 magnification.

### Statistical analyses

RBPs were considered statistically significant if they had an adjusted *p*-value <0.05. A comparison of proportions test (https://www.medcalc.org/calc/comparison_of_proportions.php) was used to determine statistical difference between the proportions of downregulated vs upregulated RBPs in OA cartilage. Two-tailed unpaired Student’s t tests were performed on the normalized counts of the top 10 downregulated and upregulated RBPs in OA cartilage (*n* = 20) compared to healthy cartilage (*n* = 18), and on TIPARP IHC quantification using GraphPad Prism (v8.4.2).

## Results

### RBPs are highly expressed and enriched in healthy cartilage

We first investigated in an RNA-seq dataset of healthy human knee cartilage (Fisch et al., 2018) the expression patterns of genes encoding RBPs (Gerstberger et al., 2014) compared to all other genes (nonRBPs), as well as TFs (Lambert et al., 2018) and lncRNAs (Derrien et al., 2012; Harrow et al., 2012). The overall expression of RBPs was significantly higher than those of the nonRBPs, TFs and lncRNAs (Figure 1A). Expression of ECM genes which are among the most highly expressed genes in cartilage was also compared to RBPs. The overall expression of RBPs was not statistically different from those of collagens and proteoglycans (Supplementary Figure S2A). However, expression of RBPs was significantly higher than those of glycoproteins, secreted factors, ECM-affiliated proteins and ECM regulators (Supplementary Figure S2A). Additionally, ∼54% of RBPs had average RNA-seq counts of greater than 100 (10^2^) but less than 1,000 (10^3^); whereas only ∼32% of TFs, ∼26% of nonRBPs and ∼12% of lncRNAs had abundances in this range (Figure 1B, Supplementary Tables S3–S6). In comparison, ∼13% of collagens, ∼31% of glycoproteins, ∼14% of proteoglycans, ∼11% of secreted factors, ∼15% of ECM-affiliated proteins and ∼21% of ECM regulators had abundances in this range (Supplementary Figure S2B; Supplementary Tables S7–S12).

We next asked which RBPs were specifically enriched in cartilage. As described in (Gamini et al., 2017; Lee et al., 2020), we compared our RNA-seq data from normal cartilage to transcriptomes from 37 tissues in the Genotype-Tissue Expression (GTEx) database. This analysis revealed 313 cartilage-enriched genes (Supplementary Table S1). Of these, 11 were RBPs (Figure 1C, Supplementary Table S13), 49 were TFs (Figure 1D, Supplementary Table S13) and 19 were lncRNAs (Figure 1E, Supplementary Table S13). In comparison, 41 were ECM genes (5 collagens, 10 glycoproteins, 7 proteoglycans, 4 secreted factors, 7 ECM-affiliated and 4 ECM regulators) (Supplementary Figures S2C–H, Supplementary Table S13). Average normalized counts of the 11 cartilage-enriched RBPs in all GTEx tissues are shown in a heatmap (Figure 1F). Further, of these 11 RBPs, TIPARP had the highest expression, which is further visualized in the box and whiskers plot (Figure 1G).

### Dysregulation of RBPs in human OA cartilage

In OA cartilage, 250 RBPs were DE compared to healthy at an adjusted *p*-value <0.05 (Supplementary Figure S3A; Supplementary Table S14). More RBPs were downregulated (153 of 250) compared to upregulated (97 of 250), and this difference was statistically significant. A similar effect was observed with TFs and lncRNAs (Supplementary Figures S3B,C; Supplementary Table S14). In contrast, the opposite trend was observed for all ECM categories—there was more upregulation than downregulation of genes encoding ECM components in OA cartilage compared to healthy (Supplementary Figures S3D–I; Supplementary Table S14).

**FIGURE 2:**
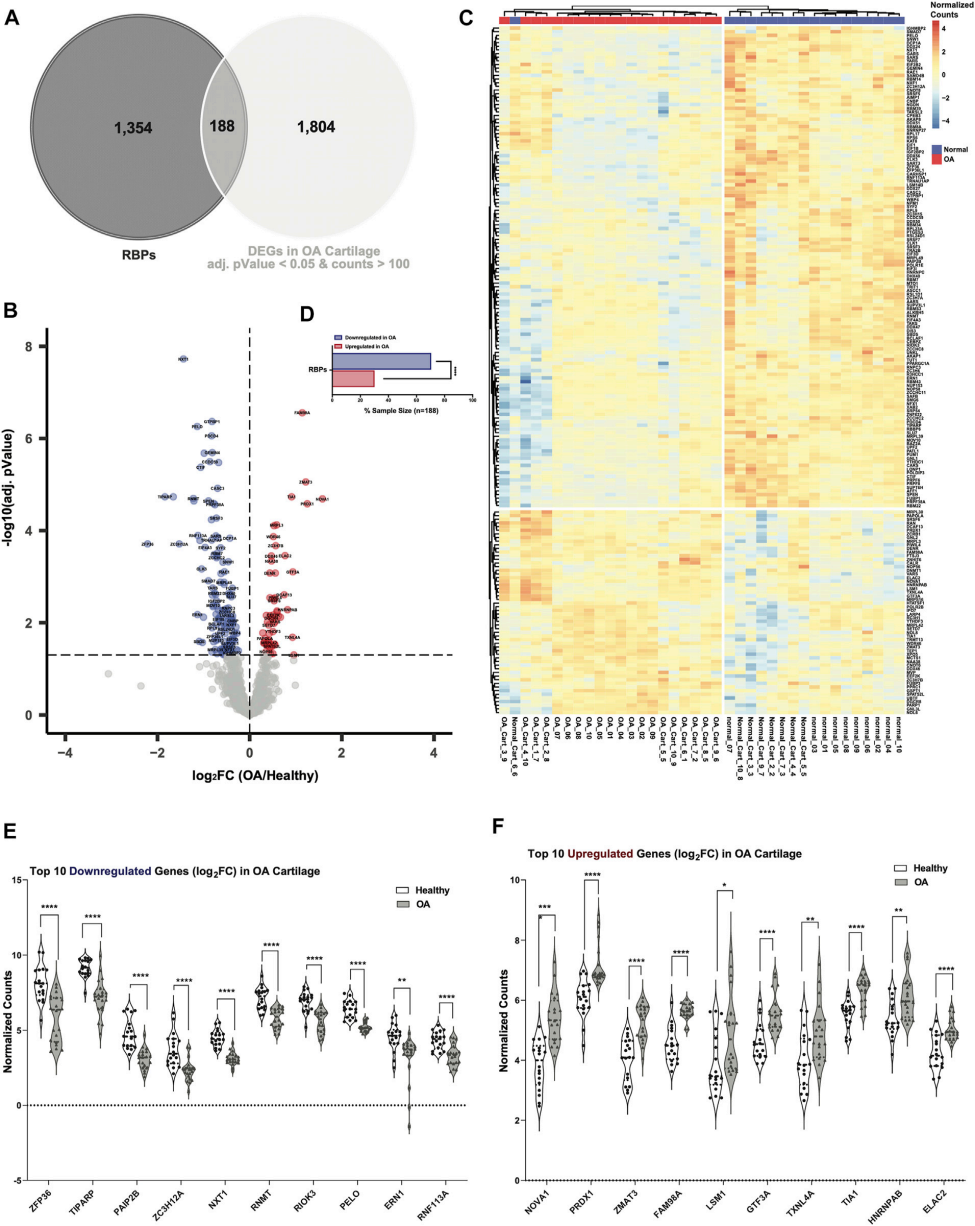
Dysregulated RBPs in OA cartilage. **(A)** Venn diagram showing intersection of RBPs with DEGs in OA cartilage with healthy RNA-seq counts >100 and an adjusted *p*-value <0.05. **(B)** Volcano plot showing 188 DE RBPs. Blue indicates downregulation in OA cartilage compared to healthy, and red indicates upregulation. Gray indicates no significance. **(C)** Heatmap showing normalized expression of the 188 DE RBPs in healthy (*n* = 18, blue) and OA (*n* = 20, red) cartilage. **(D)** Bar chart showing proportion of downregulated (blue) vs upregulated (red) RBPs in OA cartilage. ****p < 0.0001 by comparison of proportions test. **(E, F)** Violin plots showing top 10 downregulated **(E)** and upregulated **(F)** RBPs by log_2_FC. Normalized counts for each gene shown on the *y*-axes. *p < 0.05, **p < 0.01, ***p < 0.001, ****p < 0.0001 by multiple unpaired student’s t tests. For all panels, cartilage bulk RNA-seq data from (Fisch et al., 2018).

To narrow down the DE RBPs in OA cartilage, we set an RNA-seq counts threshold of >100 to potentially eliminate DE RBPs that were likely not expressed at levels sufficient for biological significance in healthy cartilage. In OA cartilage, 188 RBPs were DE compared to healthy at an adjusted *p*-value of <0.05 and also had RNA-seq counts >100 in healthy cartilage (Figure 2A, Supplementary Table S15). Dysregulated RBPs are visualized in a volcano plot (Figure 2B), and normalized counts of these DE RBPs are shown in a heatmap (Figure 2C). More RBPs were downregulated (132 of 188) in OA than upregulated (56 of 188), and this difference was statistically significant (Figure 2D). The top 10 downregulated RBPs in OA cartilage included: Zinc Finger Protein 36 (*ZFP36*)*, TIPARP,* Poly(A) Binding Protein Interacting Protein 2B (*PAIP2B*)*,* Zinc Finger CCCH-Type Containing 12A (*ZC3H12A*)*,* Nuclear Transport Factor 2 Like Export Factor 1 (*NXT1*)*,* RNA Guanine-7 Methyltransferase (*RNMT*)*,* RIO Kinase 3 (*RIOK3*)*,* Pelota mRNA Surveillance and Ribosome Rescue Factor (*PELO*)*,* Endoplasmic Reticulum to Nucleus Signaling 1 (*ERN1*) and Ring Finger Protein 113A (*RNF113A*) (Figure 2E). ZFP36 has previously been linked to OA through its regulation of SRY-Box Transcription Factor 9 (SOX9) (McDermott et al., 2016), a critical regulator of chondrogenesis. The top 10 upregulated RBPs in OA cartilage were: NOVA Alternative Splicing Regulator 1 (*NOVA1*)*,* Peroxiredoxin 1 (*PRDX1*)*,* Zinc Finger Matrin-Type 3 (*ZMAT3*)*,* Family with Sequence Similarity 98 Member A (*FAM98A*)*,* U6 snRNA-Associated Sm-Like Protein LSm1 (*LSM1*)*,* General Transcription Factor IIIA (*GTF3A*)*,* Thioredoxin Like 4A (*TXNL4A*)*,* TIA1 Cytotoxic Granule Associated RNA Binding Protein (*TIA1*)*,* Heterogeneous Nuclear Ribonucleoprotein A/B (*HNRNPAB*) and ElaC Ribonuclease Z 2 (*ELAC2*) (Figure 2F).

### Protein-protein interaction (PPI) network of the DE RBPs

After identifying the DE RBPs in OA cartilage, we investigated their protein associations using the STRING database (v11.5) (Szklarczyk et al., 2011). The 188 DE RBPs (nodes) formed a highly dense protein-protein interaction (PPI) network, with a total of 1322 interactions (edges) and an average node degree of 14.1 (Supplementary Figure S4). The STRING-defined random expected number of edges for this network was 359, so the PPI enrichment *p*-value was highly significant (<1.0e-16). The high node degree and small enrichment *p*-value suggest that the proteins in this network have a high degree of functional association, and that the associations are non-random and the observed number of edges are significant.

**FIGURE 3:**
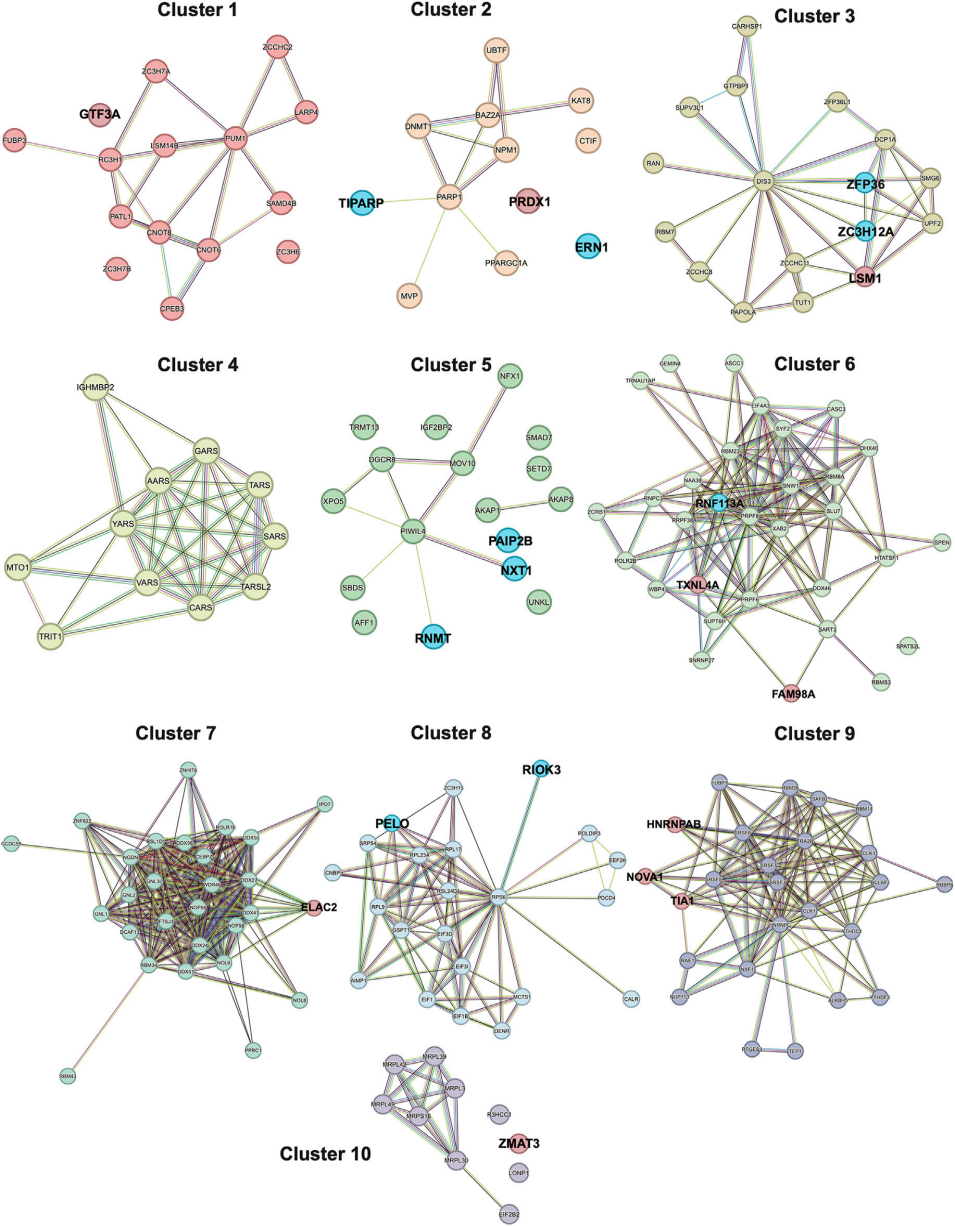
STRING PPI network of the DE RBPs, separated into 10 *k*-Means clusters. Clustering was performed using 10 clusters, which was determined using the rule of thumb (*k* = **√** *n*/2), where *n* is equal to the number of nodes (protein interactors, *n* = 188) in the network, and *k* is the number of clusters of interest. Nodes are connected by edges, with the color of the edge representing a different type of interaction annotated in the STRING database. Turquoise edges are known interactions from curated databases. Magenta edges are known interactions that were experimentally derived. Green edges are predicted interactions based on gene neighborhoods. Red edges are predicted interactions based on gene fusions. Blue edges are predicted interactions based on gene co-occurrences. Yellow edges are interactions based on text-mining. Black edges are interactions based on co-expression patterns. Lavender edges are interactions based on protein homology. The top 10 downregulated RBPs (Figure 2E) are highlighted in blue and the top 10 upregulated RBPs (Figure 2F) are highlighted in red.

To simplify this dense network, we clustered the interactors into 10 different clusters (Figure 3, Supplementary Table S16). The top 10 down- and upregulated RBPs (Figures 2E,F) were primarily represented in clusters of variable sizes. Most of the top downregulated RBPs were part of small to mid-sized clusters: 2 (12 nodes; *TIPARP* and *ERN1*), 3 (17 nodes; *ZFP36* and *ZC3H12A*), 5 (17 nodes; *PAIP2B*, *NXT1* and *RNMT*) and 8 (22 nodes; *RIOK3* and *PELO*). In contrast, several of the top upregulated RBPs were part of the largest clusters: 6 (31 nodes; *FAM98A* and *TXNL4A*) and 9 (25 nodes; *NOVA1*, *TIA1*, and *HNRNPAB*). Interestingly; the top downregulated RBPs had very few if any interactions (edges) with other RBPs; whereas, the top upregulated RBPs had a larger number of interactions. Taken together, these data could potentially indicate the most significantly depleted RBPs in OA cartilage could function more independently; whereas, the most significantly enhanced RBPs in OA cartilage could have more promiscuous interactions and function more co-dependently.

### Ribosome biogenesis was upregulated, but RNA splicing and transport were downregulated in OA cartilage

**FIGURE 4:**
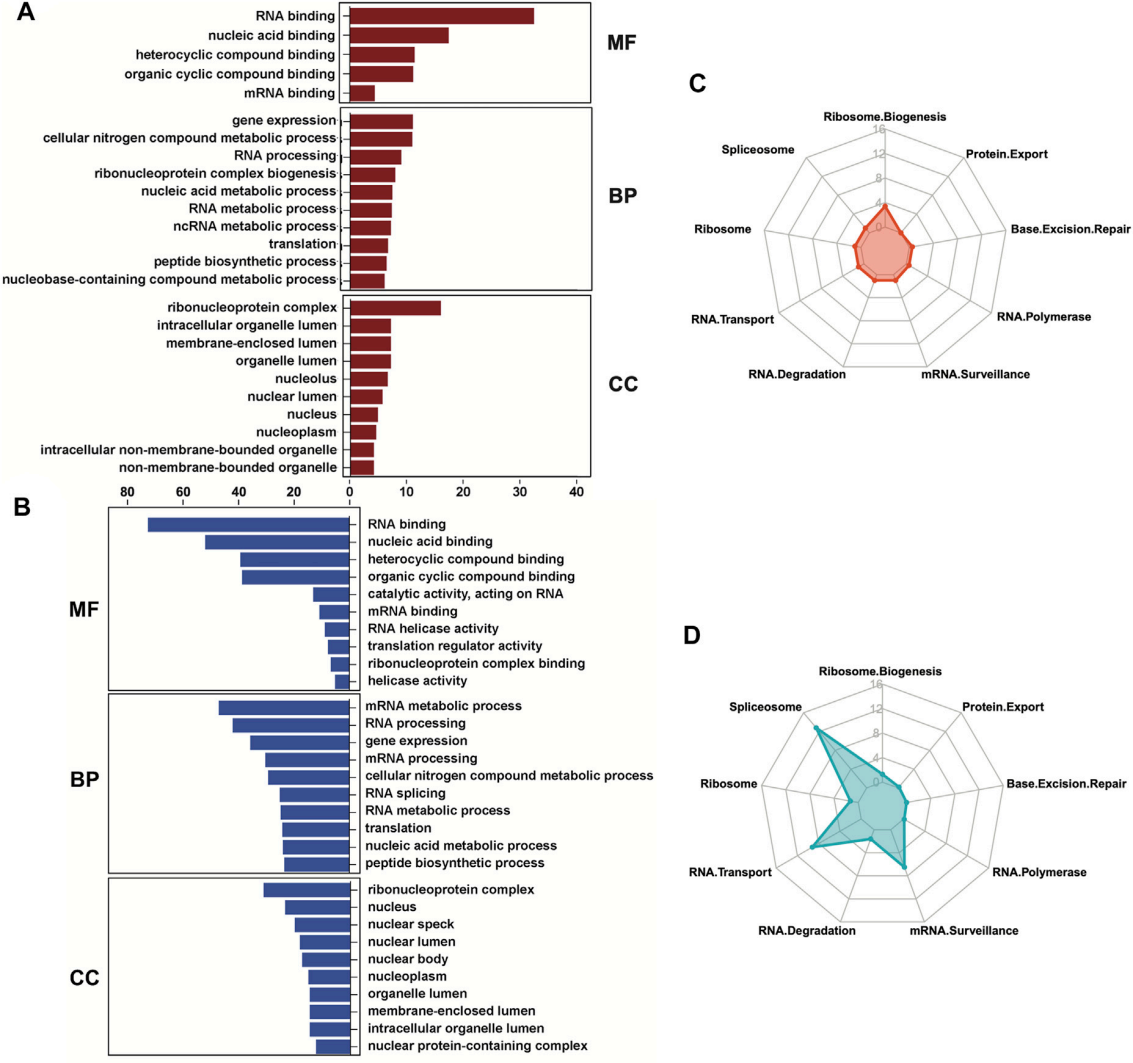
Gene ontology and biological pathways regulated by the differentially expressed RBPs. **(A, B)** Gene ontology analyses were performed on the upregulated **(A)** and downregulated **(B)** RBPs in OA cartilage using gProfiler. Molecular functions (MF), biological processes (BP) and cellular components (CC) are shown. **(C, D)** Biological pathway analyses were performed on the upregulated **(C)** and downregulated **(D)** RBPs in OA cartilage using KEGG and visualized using radar plots. –Log_10_ (adjusted *p*-value) are shown on the *y*-axes of the radar plots. For all panels, cartilage bulk RNA-seq data from (Fisch et al., 2018).

We next investigated both the gene ontology programs and biological pathways that are associated with the up- and downregulated RBPs using gProfiler (Raudvere et al., 2019). The molecular functions (MF) and cellular components (CC) of both categories were mostly overlapping, with RNA binding as the most significant MF term and ribonucleoprotein complex as the most significant CC term (Figures 4A,B). However, the biological processes (BP) differed between the up- and downregulated RBPs. Gene expression, mRNA metabolomics and RNA processing were enriched in both the up- and downregulated RBPs, but the upregulated RBPs were also enriched for ribonucleoprotein complex biogenesis (Figure 4A), while the downregulated RBPs were enriched for RNA splicing and mRNA transport (Figure 4B). Kyoto Encyclopedia of Genes and Genomes (KEGG) analyses (Kanehisa and Goto, 2000; Kanehisa, 2019; Kanehisa et al., 2023) of the biological pathways related to the DE RBPs confirmed these results. The upregulated RBPs were primarily enriched for ribosome biogenesis (Figure 4C), while the downregulated RBPs were primarily enriched for spliceosome, RNA transport and mRNA surveillance (Figure 4D).

### Transcription factors of the upregulated and downregulated RBPs

**FIGURE 5:**
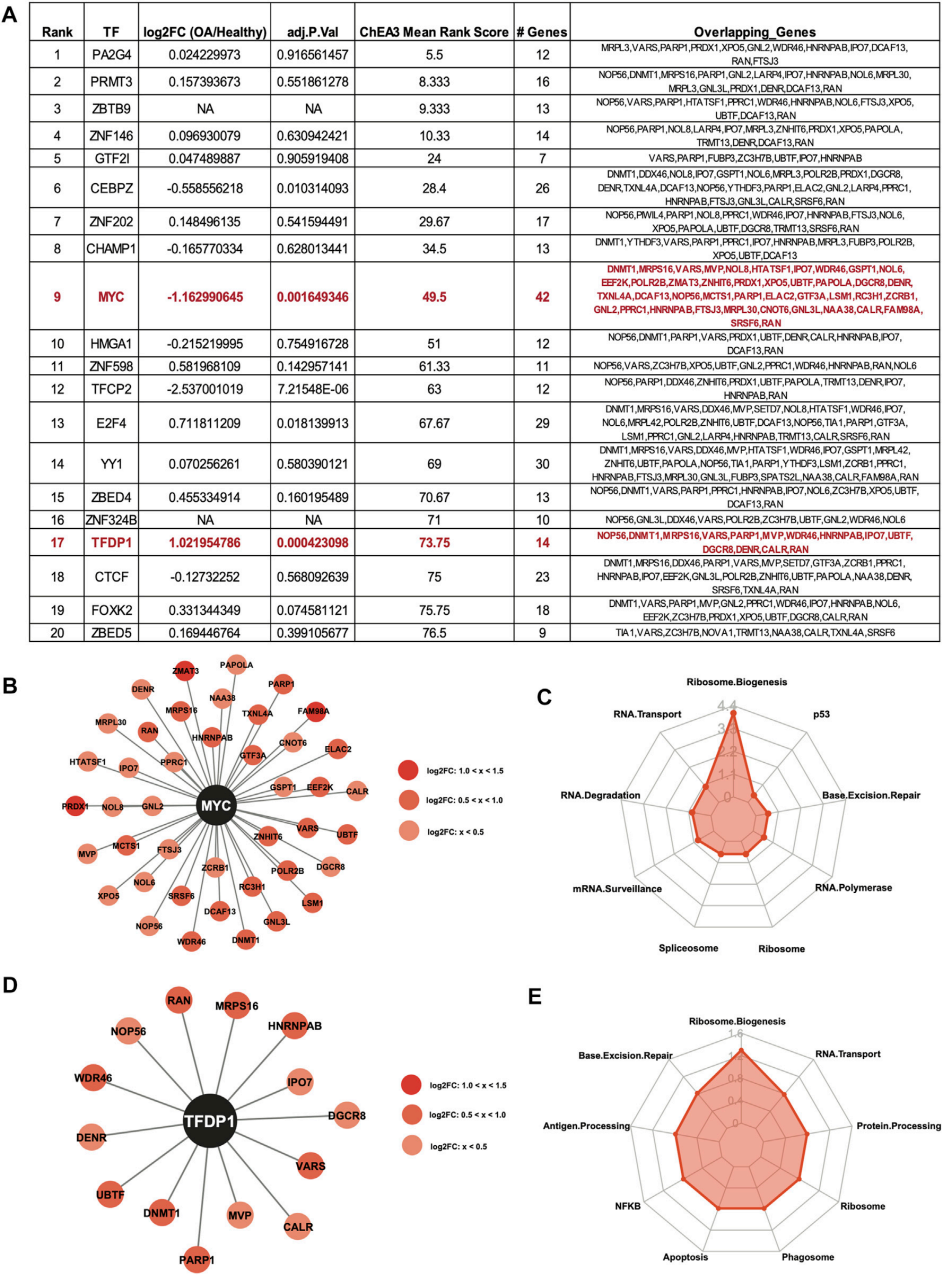
Predicted transcription factor regulators of the upregulated RBPs. **(A)** Top 20 ChEA3-predicted enriched transcription factors regulating the upregulated RBPs. TFs are ranked by Mean Rank Score. Log_2_FC and adjusted *p* values for each TF from (Fisch et al., 2018) are also included in the table. The number of genes and the gene symbols for each enriched TF are shown. MYC and TFDP1 are highlighted in red. **(B)** Cytoscape plot showing MYC and its 42 predicted upregulated RBP targets. Log_2_FC values are indicated by shade of red. **(C)** Radar plot visualizing the KEGG analysis of the 42 RBP MYC-targets. –Log_10_ (adjusted *p*-value) are shown on the *y*-axis of the radar plot. **(D)** Cytoscape plot showing TFDP1 and its 14 predicted upregulated RBP targets. Log_2_FC values are indicated by shade of red. **(E)** Radar plot visualizing the KEGG analysis of the 14 RBP TFDP1-targets. –Log_10_ (adjusted *p*-value) are shown on the *y*-axis of the radar plot.

ChIP-X Enrichment Analysis 3 (ChEA3) (Keenan et al., 2019) was used to identify potential TFs that regulate the DE RBPs. The top 20 enriched TFs for the upregulated RBPs are shown in the table, ranked by Mean Rank Score (Figure 5A). The log_2_FC and adjusted *p* values of the TFs from (Fisch et al., 2018) are also shown in the table. Of the top 20 TFs, the MYC proto-oncogene (MYC) was predicted to regulate the largest number of upregulated RBPs at 42. Interestingly, *MYC* gene expression was significantly downregulated in OA cartilage compared to healthy (Figure 5A), suggestive of a repressive role for MYC. Although the activator function of MYC is very well understood, the repressor function of this TF has also been previously reported (Li et al., 1994; Marhin et al., 1997; Wu et al., 1999; Wanzel et al., 2003). The 42 RBPs predicted to be regulated by MYC are shown in the Cytoscape plot (Figure 5B). KEGG analysis (Kanehisa and Goto, 2000; Kanehisa, 2019; Kanehisa et al., 2023) revealed the top enriched biological pathway regulated by these genes was ribosome biogenesis (Figure 5C). Additionally, Transcription Factor Dp-1 (TFDP1) was predicted to regulate 14 of the upregulated RBPs. TFDP1 itself was also significantly upregulated in OA cartilage compared to healthy, so the role of this TF in the control of the RBPs is likely as an activator. The 14 RBPs and TFDP1 are shown in a Cytoscape plot (Figure 5D). As before, KEGG analysis (Kanehisa and Goto, 2000; Kanehisa, 2019; Kanehisa et al., 2023) of the 14 RBPs revealed enrichment for ribosome biogenesis (Figure 5E).

**FIGURE 6:**
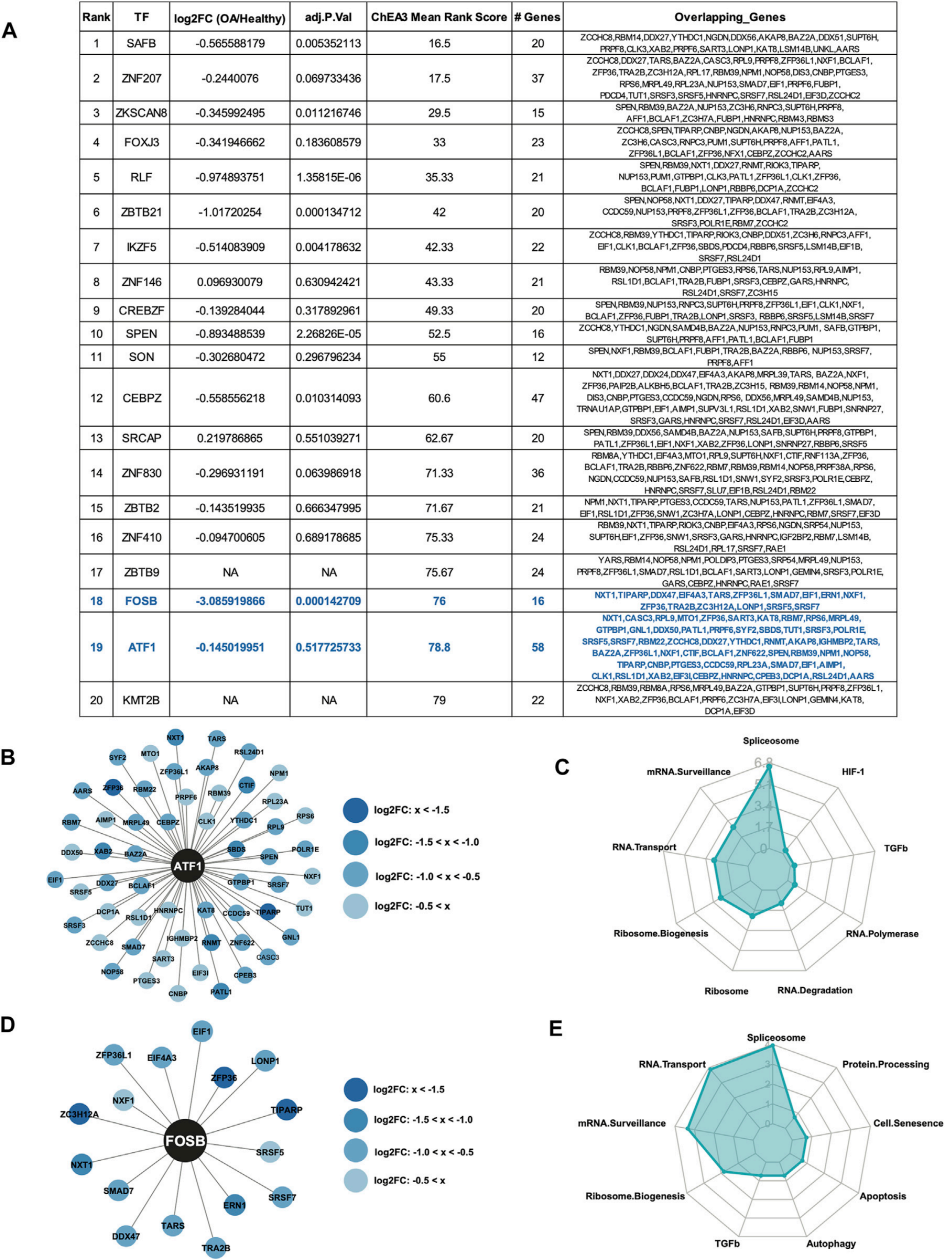
Predicted transcription factor regulators of the downregulated RBPs. **(A)** Top 20 ChEA3-predicted enriched transcription factors regulating the downregulated RBPs. TFs are ranked by Mean Rank Score. Log_2_FC and adjusted *p* values for each TF from (Fisch et al., 2018) are also included in the table. The number of genes and the gene symbols for each enriched TF are shown. ATF1 and FOSB are highlighted in blue. **(B)** Cytoscape plot showing ATF1 and its 58 predicted downregulated RBP targets. Log_2_FC values are indicated by shade of blue. **(C)** Radar plot visualizing the KEGG analysis of the 58 RBP ATF1-targets. –Log_10_ (adjusted *p*-value) are shown on the *y*-axis of the radar plot. **(D)** Cytoscape plot showing FOSB and its 16 predicted downregulated RBP targets. Log_2_FC values are indicated by shade of blue. **(E)** Radar plot visualizing the KEGG analysis of the 16 RBP FOSB-targets. –Log_10_ (adjusted *p*-value) are shown on the *y*-axis of the radar plot.

The top 20 enriched TFs for the downregulated RBPs are shown in the table, ranked by Mean Rank Score (Figure 6A). Of the top 20 TFs, Activating Transcription Factor 1 (ATF1) was predicted to regulate the largest number of downregulated RBPs at 58. The 58 RBPs predicted to be regulated by ATF1 are shown in the Cytoscape plot (Figure 6B). KEGG analysis (Kanehisa and Goto, 2000; Kanehisa, 2019; Kanehisa et al., 2023) revealed the top enriched biological pathway regulated by these genes was spliceosome (Figure 6C). Additionally, FosB proto-oncogene (FOSB) was predicted to regulate 16 of the downregulated RBPs. FOSB itself was also significantly downregulated in OA cartilage compared to healthy, so the role of this TF in the control of the RBPs is likely as an activator. The 16 RBPs and FOSB are shown in a Cytoscape plot (Figure 6D). As before, KEGG analysis (Kanehisa and Goto, 2000; Kanehisa, 2019; Kanehisa et al., 2023) of the 16 RBPs revealed enrichment for spliceosome, mRNA transport and mRNA surveillance (Figure 6E).

### TIPARP as a candidate therapeutic target in OA cartilage

**FIGURE 7:**
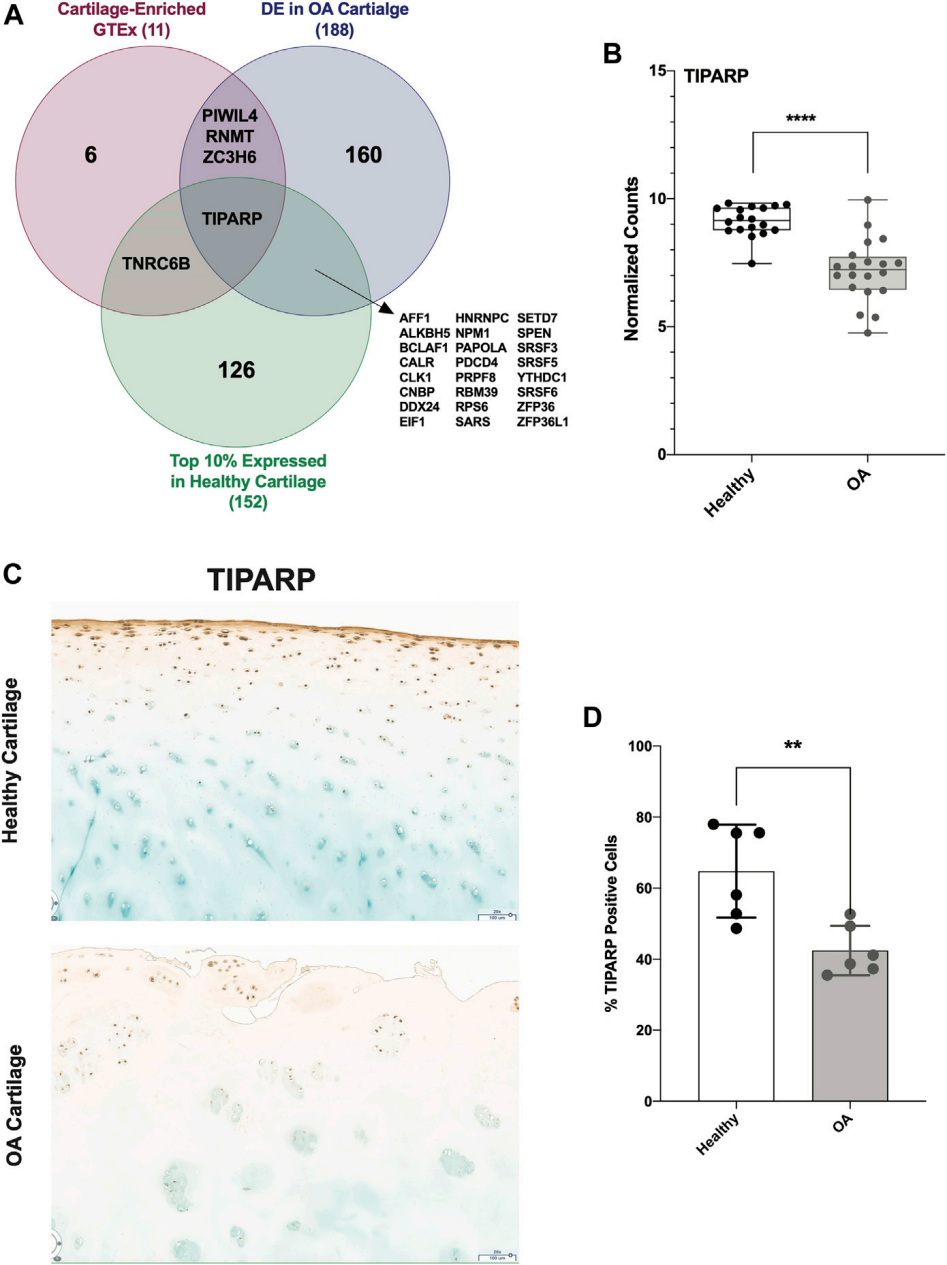
TIPARP as a novel candidate therapeutic target in OA cartilage. **(A)** Venn diagram showing intersection of highly expressed (top 10%) RBPs in healthy cartilage, GTEx cartilage-enriched RBPs and DE RBPs in OA cartilage. **(B)** Box plot showing TIPARP normalized counts from healthy (*n* = 18) and OA (*n* = 20) cartilage samples. ****p < 0.0001 by unpaired Student’s t-test. For all panels, cartilage bulk RNA-seq data from (Fisch et al., 2018). **(C, D)** IHC **(C)** and quantification **(D)** of TIPARP protein in OA compared to healthy in articular cartilage. Error bars are standard deviation, *n* = 6 (healthy) and *n* = 6 (OA). *p<0.05 by two-tailed unpaired Student’s t-test.

To identify a candidate RBP for further studies and as potential therapeutic targets, we investigated other parameters in addition to differential expression. First, we identified the top 10% expressed RBPs in healthy cartilage. Next, we identified those that were cartilage-enriched according to analysis of GTEx. Only 11 RBPs were cartilage-enriched, 4 of which were DE in OA cartilage. Intersecting these 3 criteria, we identified TIPARP as a candidate RBP for further investigation (Figure 7A). *TIPARP* expression was significantly reduced in OA compared to healthy (Figure 7B). TIPARP protein was also significantly depleted in OA cartilage compared to healthy as shown by IHC analysis (Figures 7C,D).

### scRNA-seq data reveal cluster-specific expression patterns of TIPARP in healthy and OA cartilage

Our analyses thus far have utilized our previously published bulk RNA-seq data from healthy (*n* = 18) and OA (*n* = 20) cartilage samples (Fisch et al., 2018). Although useful for a broad view of disease impact, bulk RNA-seq data only reveal average changes in gene expression across tissues that are often highly heterogeneous in cell-type composition. Disease-related changes restricted to certain cell subpopulations would be masked or even lost entirely. Single-cell RNA sequencing (scRNA-seq) technologies overcome these limitations by capturing global gene expression profiles at a single-cell resolution.

**FIGURE 8:**
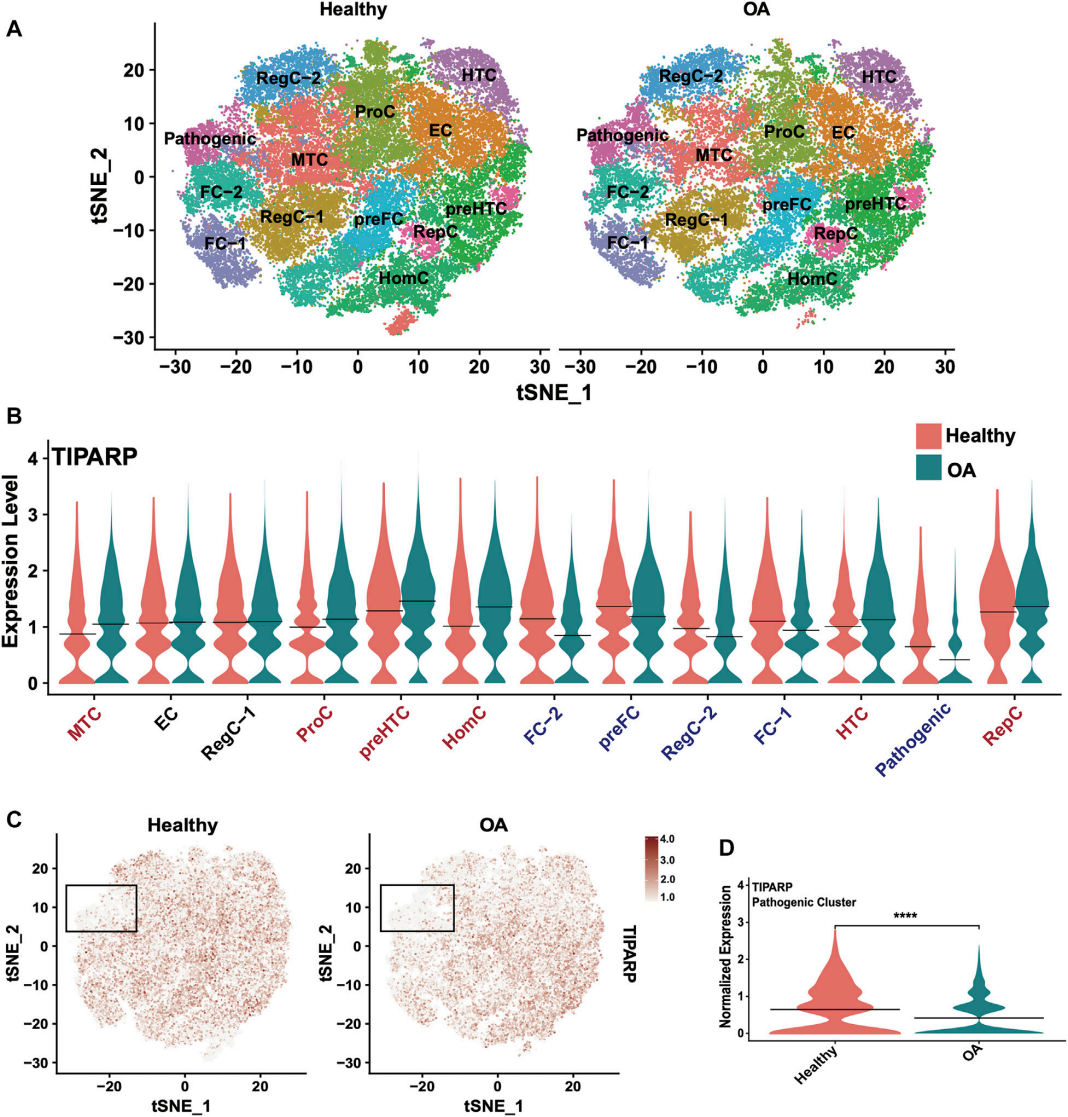
Single-cell RNA sequencing reveals differential *TIPARP* expression patterns in healthy and OA human cartilage. **(A)** Visualization of clustering by split tSNE plots of healthy (*n* = 6) and OA (*n* = 6) human cartilage samples using 0.5 resolution. **(B)** Violin plots showing cluster-specific *TIPARP* expression split by condition (healthy [pink] vs OA [blue]). *TIPARP* is upregulated in OA in clusters with red text, downregulated in OA in clusters with blue text, and unchanged in clusters with black text. **(C)** *TIPARP* expression in healthy vs OA cartilage visualized in split tSNE plots. The pathogenic cluster is indicated with black boxes. Normalized expression is shown in the color-coded scale. **(D)** Differential expression of *TIPARP* between healthy [pink] and OA [blue] in the pathogenic cluster. Black lines at the mean. ****p < 0.0001 by unpaired Student’s t-test. For all panels: RegC: regulatory chondrocytes, EC: effector chondrocytes, preFC: pre-fibrocartilage chondrocytes, FC: fibrocartilage chondrocytes, preHTC: pre-hypertrophic chondrocytes, HTC: hypertrophic chondrocytes, HomC: homeostatic chondrocytes and RepC: reparative chondrocytes. For all panels, cartilage scRNA-seq data from (Swahn et al., 2023).

To expand our investigation of TIPARP, we interrogated cluster-specific, disease-related changes in *TIPARP* expression using our scRNA-seq data from healthy (*n* = 6) and OA (*n* = 6) cartilage (Swahn et al., 2023). The published cell subsets in healthy and OA cartilage are shown in the t-distributed stochastic neighbor embedding (tSNE) plot (Figure 8A). Our scRNA-seq data revealed differential expression of *TIPARP* in cell subpopulations. In the most chondrocytic clusters (preHTC and HTC), TIPARP mRNA was enhanced in OA compared to healthy (Figure 8B). In contrast, in the most fibrocytic clusters (preFC, FC-1 and FC-2), TIPARP mRNA was depleted in OA compared to healthy (Figure 8B). Most notably, *TIPARP* expression was significantly downregulated in the cell subset that was termed “pathogenic cluster”, as it was enriched for the expression of genes that promote OA pathogenesis (Swahn et al., 2023) (Figures 8B–D). Thus, although overall *TIPARP* expression was reduced in OA, this reduction appeared to be more pronounced in the fibrocytic and “pathogenic” clusters found in cartilage. Taken together, these data suggest a context-specific effect for TIPARP in chondrocyte function, which was not evident in the bulk RNA-seq data alone.

## Discussion

Our global analyses offered insight into expression patterns of RBPs in healthy cartilage, as well as a comprehensive view of dysregulated RBPs in OA cartilage. RBPs were shown to be highly expressed in healthy cartilage, particularly when compared to genes encoding other classes of regulatory factors such as TFs and lncRNAs. Similar expression patterns were also observed in cancer tissues, with genes encoding RBPs more highly expressed than nonRBPs, TFs, lncRNAs and microRNAs (Kechavarzi and Janga, 2014). Our analyses uncovered several RBPs that are highly enriched in cartilage as compared to a broad spectrum of other human tissues, suggesting the hypothesis that they are involved in cartilage development, in stem cells undergoing chondrogenesis and in the maintenance of the phenotype of differentiated chondrocytes.

Global dysregulation of RBPs has been observed in other diseases, particularly cancers (Kechavarzi and Janga, 2014; Mukherjee and Goswami, 2021). We identified 188 DE RBPs in OA cartilage (∼12% of all RBPs expressed in healthy cartilage). A larger proportion of RBPs were downregulated compared to upregulated in OA, indicative of significant dysregulation of post-transcriptional processes. The upregulated RBPs were primarily enriched for ribosome biogenesis. Translational defects as a result of ribosome biogenesis dysregulation have been previously reported in OA cartilage—particularly during the late-stage of disease—and OA has been hypothesized to be an acquired ribosomopathy (van den Akker et al., 2022). In contrast to the upregulated RBPs, the downregulated RBPs in OA cartilage were primarily enriched for spliceosome, RNA transport and mRNA surveillance. Dysregulation in alternative splicing in OA cartilage has previously been demonstrated. One example is fibronectin-1 (*FN1*). The canonical, full-length isoform of *FN1* was shown to be downregulated in OA cartilage, but a truncated isoform—*FN1-208*—was significantly upregulated (van Hoolwerff et al., 2023). Alterations in the ratio of *FN1* to *FN1-208* resulted in downregulation of critical chondrocytic genes Aggrecan (*ACAN*) and Collagen Type II Alpha 1 Chain (*COL2A1*).

Our analyses thus far identified RBPs as intriguing candidates for therapeutics; however, it is often challenging to prioritize and select specific candidates with greater potential for therapeutic targeting. For this reason, we intersected three different criteria: (i) the 188 DE RBPs in OA cartilage, (ii) the top 10% of RBPs expressed in healthy cartilage and (iii) the 11 that were cartilage-enriched according to analysis of GTEx data. This approach identified TIPARP as a promising novel candidate therapeutic target in OA cartilage. This RBP was the only RBP DE in OA cartilage compared to healthy, highly expressed in healthy cartilage and cartilage-enriched. TIPARP is a member of the PARP family of proteins, and is an ADP-ribosyltransferase that mediates mono-ADP ribosylation of glutamate, aspartate and cysteine residues on target proteins (MacPherson et al., 2013; Vyas et al., 2014; Gomez et al., 2018). TIPARP contains a zinc finger domain, which is its putative RNA-binding domain (Schreiber et al., 2006; Cook et al., 2011). *TIPARP* expression was significantly downregulated in OA cartilage in our data, consistent with prior reports (Soul et al., 2018; Zhang et al., 2018), using bulk RNA-seq datasets, which average gene expression across all cell subsets present in a tissue type. Mining our scRNA-seq dataset of healthy and OA cartilage, we were able to interrogate the cluster-specific expression patterns of *TIPARP*. We observed enhancement of *TIPARP* in OA compared to healthy in the chondrocytic clusters (i.e., preHTC, HTC and RepC), but depletion of *TIPARP* in OA compared to healthy in the fibrocytic clusters (i.e., preFC, FC-1 and FC-2). Additionally, we observed significant suppression of *TIPARP* in the “pathogenic” cell cluster, which further suggests a functional linkage with OA.

Although TIPARP as a candidate therapeutic target in OA cartilage is novel, this RBP has been suggested as a therapeutic target in breast cancer. TIPARP was shown to downregulate oncogenic transcription factors, and its interacting partners were enriched for telomere maintenance and organization (Cheng et al., 2019; Zhang et al., 2020). Additionally, *TIPARP* expression can be induced by metformin (Cheng et al., 2019) which is in clinical use for the treatment of diabetes (Nasri and Rafieian-Kopaei, 2014). Metformin also has anti-inflammatory and anti-aging properties, mediated in part via AMPK signaling (Salminen and Kaarniranta, 2012; Wang et al., 2020), and has been proposed as a disease modifying therapy for OA (Lim et al., 2022). Metformin has been suggested to attenuate OA through prevention of cartilage destruction and reduction of pain (Li et al., 2020; Lim et al., 2022; Song et al., 2022). TIPARP could potentially play a role in these mechanisms, although functional studies would be required to validate this hypothesis.

In addition to TIPARP, we revealed several other RBPs of prime interest including ZFP36 and TIA1. *ZFP36* was the most significantly downregulated RBP by log_2_FC in OA cartilage (Figure 2E), and *TIA1* was in the top 10 upregulated RBPs (Figure 2F). ZFP36 was also in the top 10% of expressed RBPs in healthy cartilage (Figure 7A). Both of these RBPs have been reported previously as influencing OA pathogenesis (Ansari and Haqqi, 2016; McDermott et al., 2016). Identifying these RBPs in our own data analyses gives confidence to our pipeline, as well as RBPs being a potentially appealing class of proteins for therapeutic targeting.

This study has limitations. First, we used an RNA-seq counts cutoff of >100 in healthy cartilage. This could potentially bias our analysis and filter out RBPs with low copy numbers or restricted expression to subsets of cells. There were 62 DE RBPs that did not meet our RNA-seq counts threshold (Supplementary Table S17). Additionally, our analyses were only based on transcriptomic data. Inclusion of data on protein levels and posttranslational modifications would provide more accurate predictions on RBP functions. Besides cartilage, OA affects other tissues and knowledge of RBPs in those tissues would help to better predict RBPs as therapeutic targets. Although TIPARP is a promising novel candidate for OA therapeutics, our bioinformatic predictions must be validated through functional studies, so the precise mechanism of action and biological significance of TIPARP in OA cartilage can be identified.

In conclusion, our analyses offer a global view of RBP expression patterns in healthy and OA cartilage and bioinformatic predictions of top candidates that are worthy of further investigations for their role in cartilage biology and OA pathogenesis. ‘RBP’-eutics is an emerging field in which RBPs are being explored as therapeutic candidates in cancer treatments (Mohibi et al., 2019), and the global view and prioritization of RBPs from the present study provides an application of this field to OA.

## Data Availability

The original contributions presented in the study are included in the article/Supplementary Material, further inquiries can be directed to the corresponding author.
