# Supplementary figures and images for: RNA-binding proteins that are highly expressed and enriched in healthy cartilage but suppressed in osteoarthritis
Source: Front Cell Dev Biol. 2023 Jun 30;11:1208315. doi: 10.3389/fcell.2023.1208315 (PMC10349536; doi:10.3389/fcell.2023.1208315)

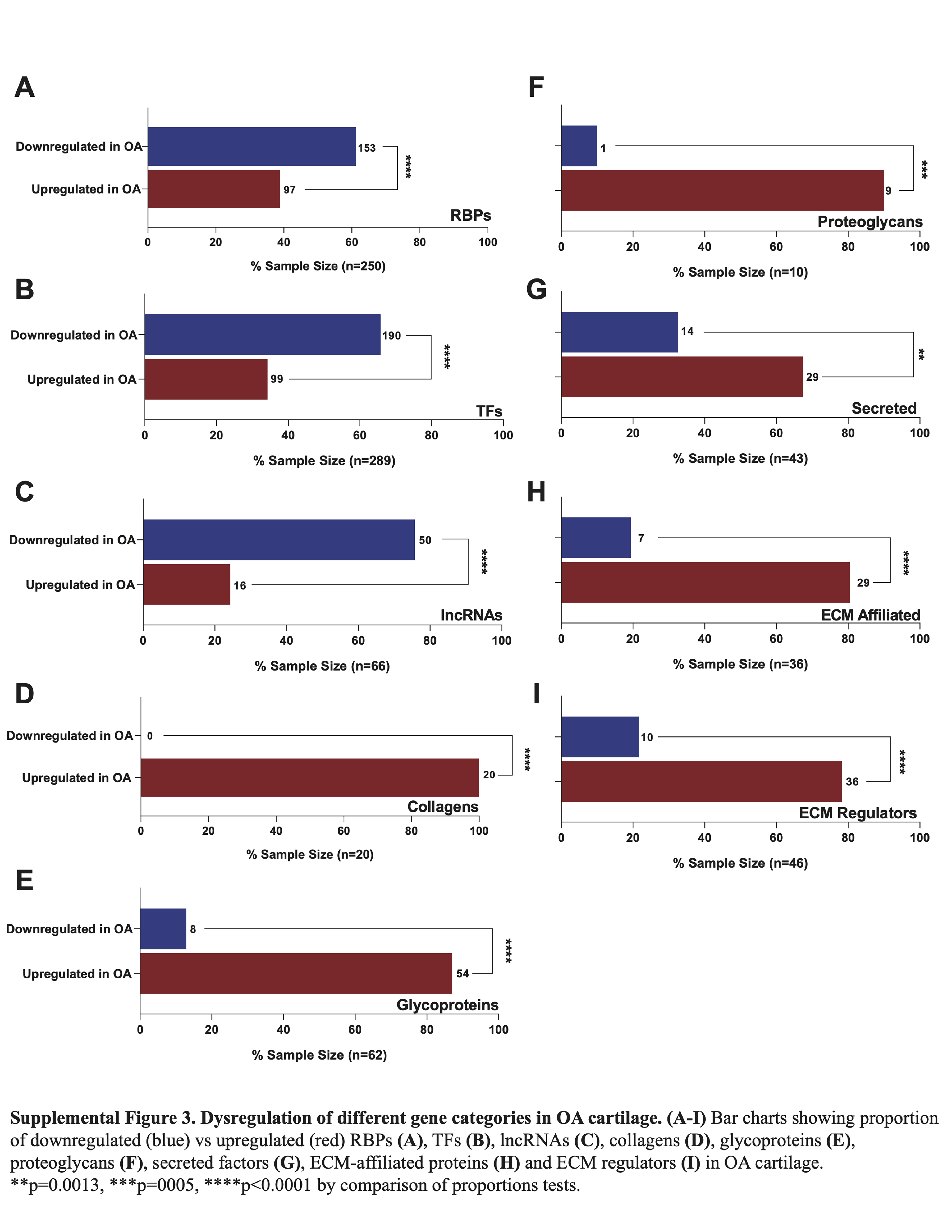

Supplement: Supplementary file 1 [file Image3.TIF]

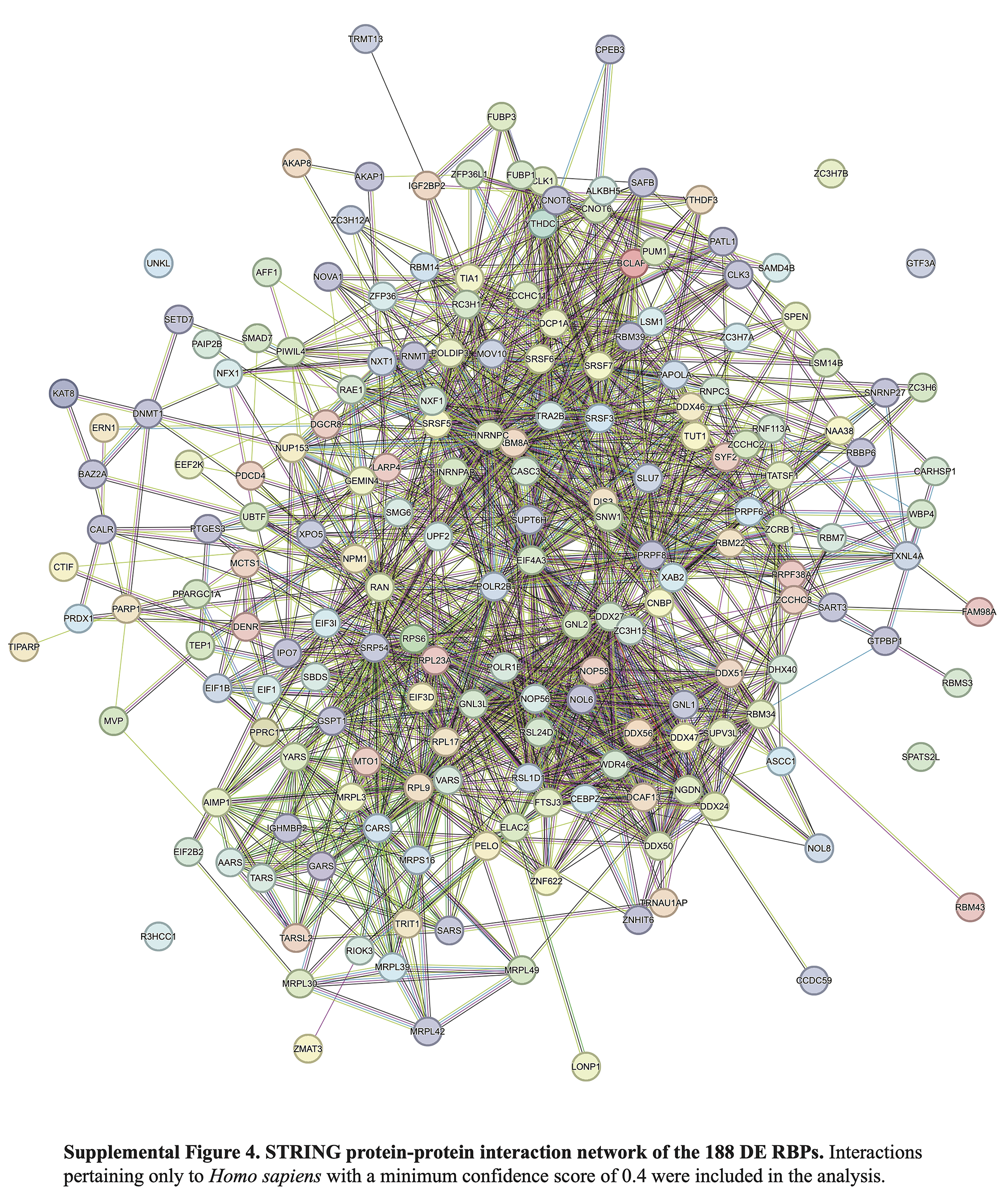

Supplement: Supplementary file 2 [file Image4.TIF]

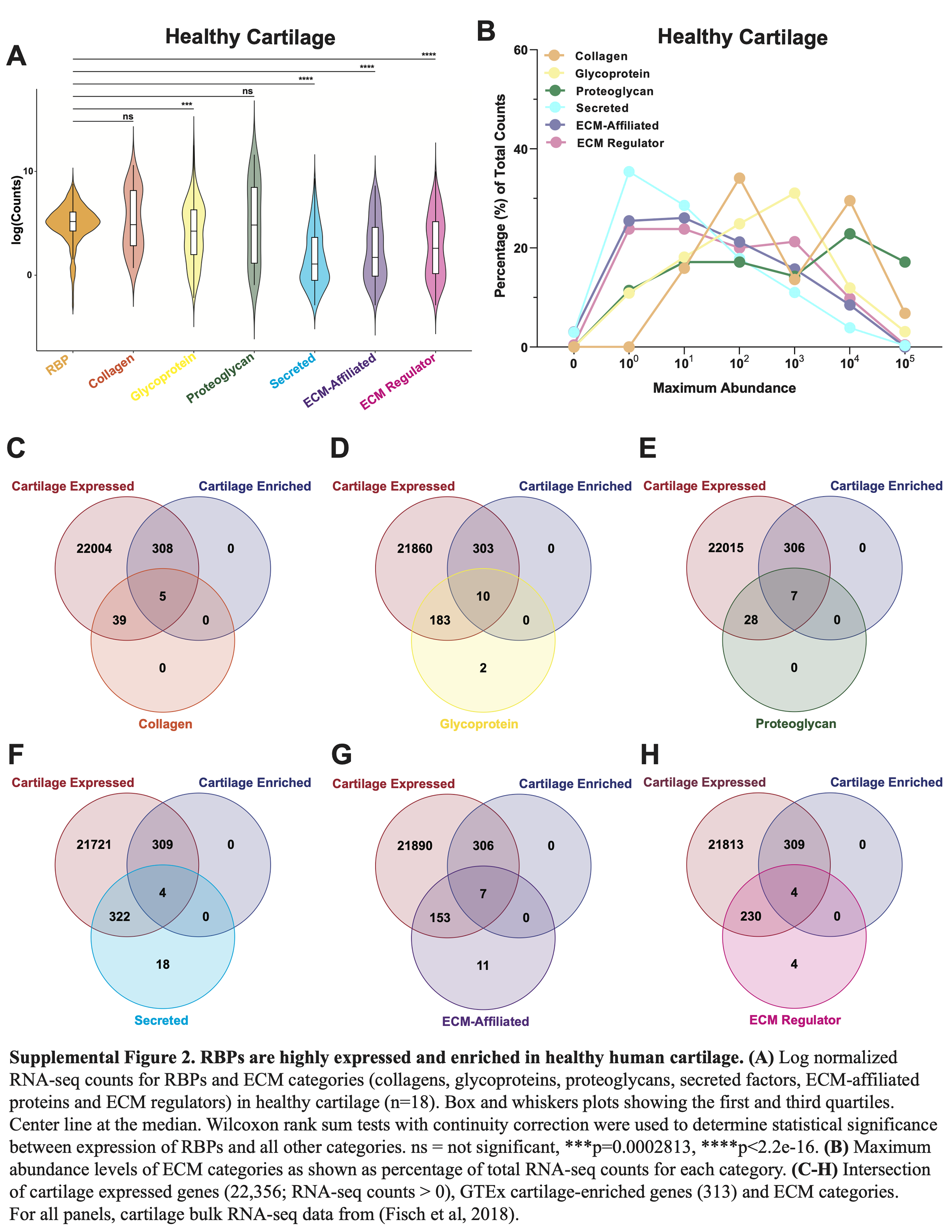

Supplement: Supplementary file 4 [file Image2.TIF]

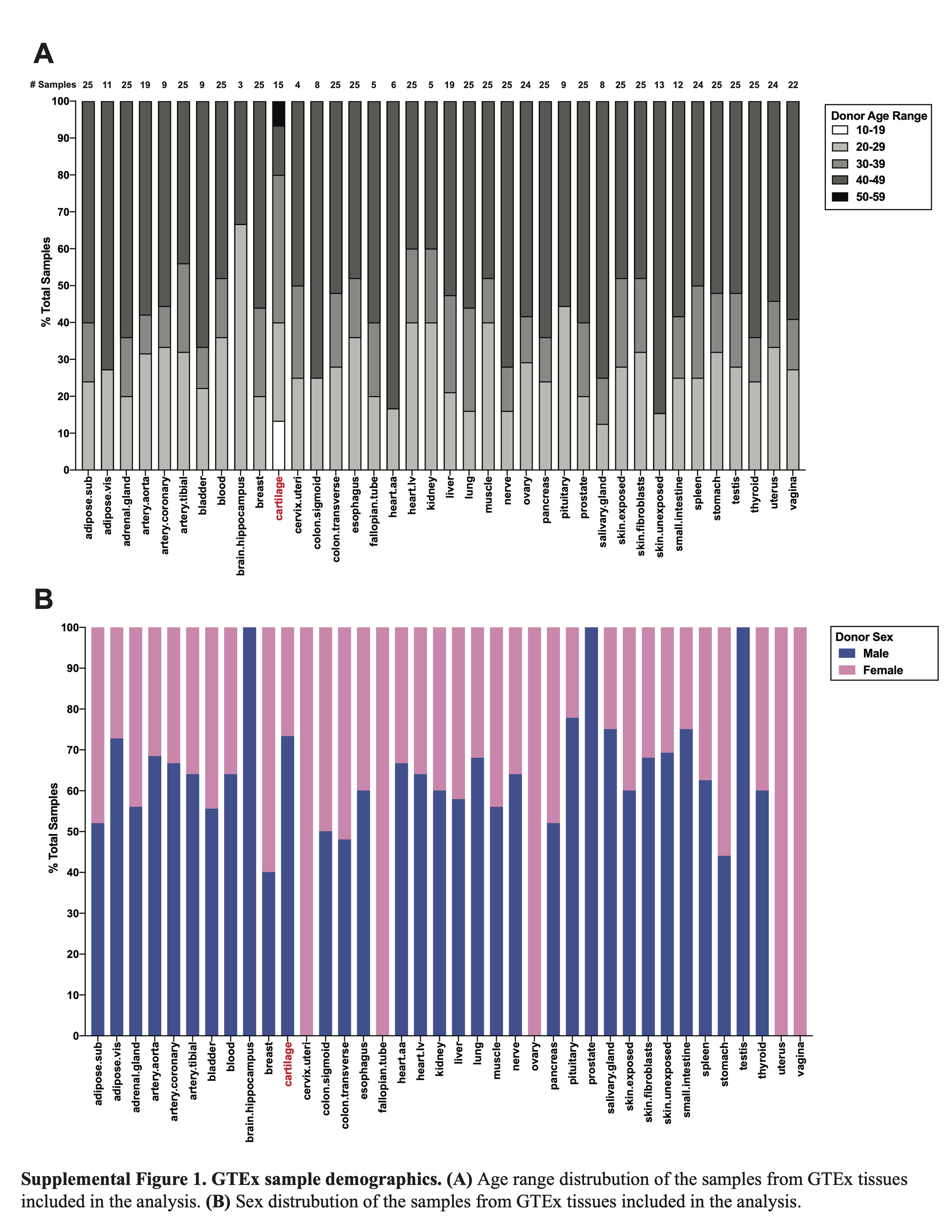

Supplement: Supplementary file 5 [file Image1.TIF]
